# *MnASI1* Mediates Resistance to *Botrytis cinerea* in Mulberry (*Morus notabilis*)

**DOI:** 10.3390/ijms232113372

**Published:** 2022-11-02

**Authors:** Donghao Wang, Na Gong, Chaorui Liu, Suxia Li, Zhaocheng Guo, Gefan Wang, Qiqi Shang, Dongming Wang, Xianling Ji, Youchao Xin

**Affiliations:** 1 College of Forestry, Shandong Agricultural University, Tai’an 271018, China; 2 State Key Laboratory of Crop Biology, Shandong Agricultural University, Tai’an 271018, China

**Keywords:** a-amylase/subtilisin inhibitor, *B. cinerea*, mulberry, plant–pathogen interaction, *MnASI1*

## Abstract

Six α-amylase/subtilisin inhibitor genes (*MnASIs*) were identified from mulberry (*Morus notabilis*). In this study, bioinformatics and expression pattern analysis of six *MnASIs* were performed to determine their roles in resistance to *B. cinerea*. The expression of all six *MnASIs* was significantly increased under *Botrytis cinerea* infection. *MnASI1*, which responded strongly to *B. cinerea*, was overexpressed in *Arabidopsis* and mulberry. The resistance of *Arabidopsis* and mulberry overexpressing *MnASI1* gene to *B. cinerea* was significantly improved, the catalase (CAT) activity was increased, and the malondialdehyde (MDA) content was decreased after inoculation with *B. cinerea*. At the same time, H_2_O_2_ and O_2_^−^ levels were reduced in *MnASI1* transgenic *Arabidopsis*, reducing the damage of ROS accumulation to plants. In addition, *MnASI1* transgenic *Arabidopsis* increased the expression of the salicylic acid (SA) pathway-related gene *AtPR1*. This study provides an important reference for further revealing the function of α-amylase/subtilisin inhibitors.

## 1. Introduction

Plant protease inhibitors (PIs) are a class of small proteins with various biological functions. They regulate endogenous protease activity and apoptosis while protecting plants from animals, insects, and microorganisms [1,2,3]. PIs are divided into different families based on their identical cysteine pattern, overall 3D structure, and mechanism of action. They can also be grouped based on amino acid sequence similarity [4,5]. There are more than 10 different families of PIs from many plant species depending on the type of protease they act on [6,7,8]. Kunitz-type protease inhibitors (PKPIs) are usually present in the storage tissues of plants and can resist pathogen infection [9]. PKPIs have a molecular weight of about 20 to 22 kDa and may consist of one to two polypeptide chains linked by one to two disulfide bonds [5]. Their structures may be missing cysteine residues [10,11]. As a member of PKPIs, α-amylase/subtilisin inhibitors (ASIs) inhibit α-amylase in mammals and lepidopteran pests [12]. Therefore, ASIs can protect plants from lepidopteran pests. In addition, ASIs have an inhibitory effect on subtilisin and are therefore also associated with plant resistance to microorganisms [2].

Mulberry (*Morus* L.) is widely distributed all over the world and has important economic value in the production of food and medicine due to its rich content of secondary metabolites that are beneficial to humans [13,14,15,16]. *Botrytis cinerea* is a necrotizing fungal pathogen that can infect more than 200 species of plants around the world [17,18,19]. At the same time, *B. cinerea* is one of the main pathogenic fungi of mulberry [20,21]. Previous studies on ASIs mainly focused on identification and classification. Although it was found to be related to plant disease resistance, the mechanism of disease resistance was not completely clear. At the same time, there is no systematic study on the role of ASIs in mulberry resistance. The aim of this study is to reveal the underlying mechanism of disease resistance in mulberry ASIs.

The availability of transcriptomic data from mulberry (*Morus notabilis*) in response to *B. cinerea* infection facilitates studies on resistance of ASIs to *B. cinerea* infection [22]. Based on the previous transcriptome data, we found that the expression levels of six ASIs in *M. notabilis* were significantly increased during *B. cinerea* infection. The resistance of ASIs to *B. cinerea* infection was investigated. Furthermore, to investigate their functions, we performed *MnASI1* expression in *Arabidopsis* and mulberry. The resistance of transgenic *Arabidopsis* and mulberry was studied by various methods, and it was confirmed that *MnASI1* was involved in the defense response of transgenic plants. These results initially revealed the mechanism of ASIs disease resistance, laid the foundation for further understanding the function of ASIs, provided a reference for other plant ASI research, and provide potential target genes for enhancing the resistance of mulberry to *B. cinerea*.

## 2. Results

### 2.1. Bioinformatics Analyses of MnASIs

A total of six α-amylase/subtilisin inhibitor genes were identified from the mulberry genome sequence. The six α-amylase/subtilisin inhibitor proteins ranged in length from 191 (*MnASI1* and *MnASI5*) to 207 amino acids (aa) (*MnASI3*) (Table 1). The relative molecular mass ranged from 20.82 kDa (*MnASI1*) to 22.65 kDa (*MnASI3*). The theoretical isoelectric points (pI) ranged from 4.46 (*MnASI1*) to 8.53 (*MnASI6*).

**Figure 1:**
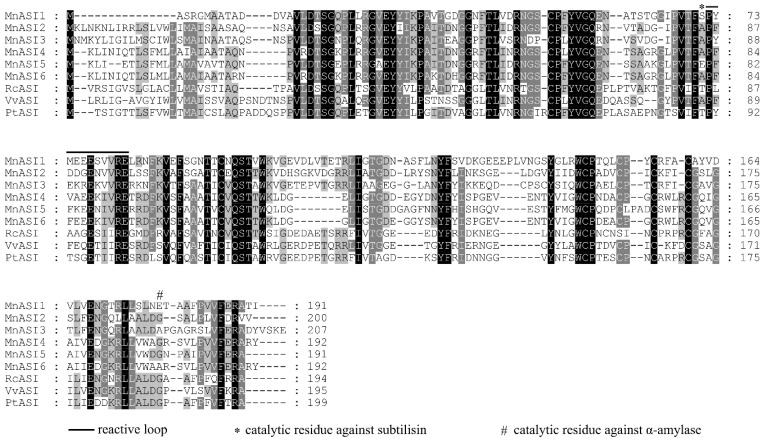
Multiple sequence alignment of *MnASIs* with other α-amylase/subtilisin inhibitors. A solid line indicates the reactive loop. An asterisk shows the catalytic residue against subtilisin. A pound sign shows the catalytic residue against α-amylase. The accession numbers obtained from GenBank are as follows: *RcASI* (*Ricinus communis*, XP_002525871), *VvASI* (*Vitis vinifera*, XP_002265535), and *PtASI* (*Populus trichocarpa*, XP_006383817).

The multiple alignment results of *MnASIs* and other plant α-amylase/subtilisin inhibitors showed that there were two regions of plant α-amylase/subtilisin inhibitors, namely the protease inhibitory region and the α-amylase inhibitory region (Figure 1).

**Figure 2:**
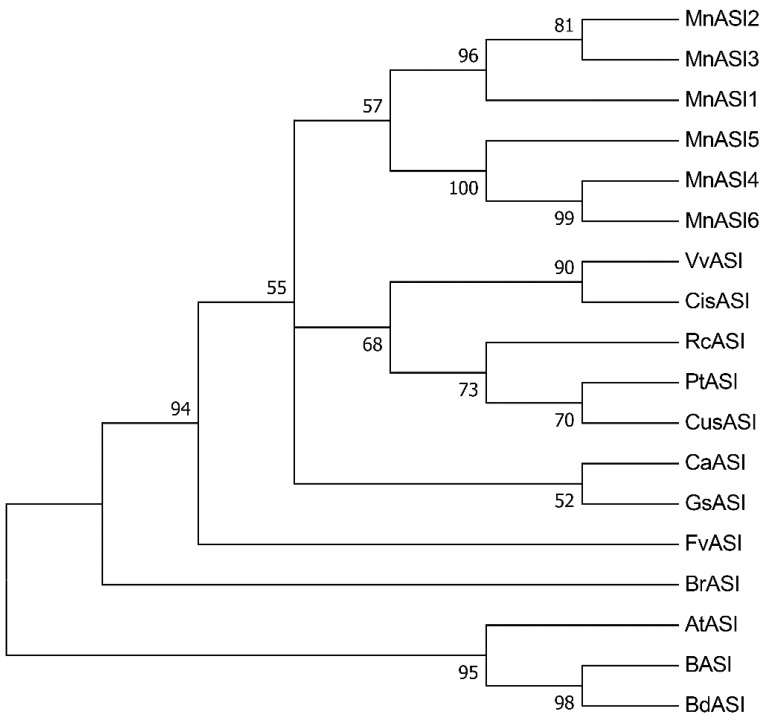
Phylogenetic tree of ASI amino acid sequences in mulberry and other plants. The tree is constructed using the neighbor-joining method. The bootstrap value is expressed as a percentage. The accession numbers obtained from GenBank are as follows: *VvASI* (*Vitis vinifera*, XP_002265535), *CisASI* (*Citrus sinensis*; XP_006468645), *RcASI* (*Ricinus communis*, XP_002525871), *PtASI* (*Populus trichocarpa*, XP_006383817), *CusASI* (*Cucumis sativus*; XP_004139193), *CaASI* (*Cicer arietinum*; XP_004514494), *GsASI* (*Glycine soja*; KHN19473), *FvASI* (*Fragaria vesca* subsp. *Vesca*; XP_004295670), *BrASI* (*Brassica rapa*; XP_009128557), *AtASI* (*Aegilops tauschii*; EMT21954.1), *BASI* (*Hordeum vulgare*; P07596.2), and *BdASI* (*Brachypodium distachyon*; XP_003581446).

The protein sequences of other plant α-amylase/subtilisin inhibitors obtained from NCBI were multiple aligned. Phylogenetic and molecular evolutionary analyses were performed using MEGA 6 to explore the evolutionary relationships among different species (Figure 2). The results showed that the *MnASIs* proteins of mulberry clustered together and were more distantly related to the α-amylase/subtilin inhibitors in *Aegilops tauschii*, *Hordeum vulgare*, and *Brachypodium distachyon*, and were more closely related to the α-amylase/subtilin inhibitors in *Vitis vinifera* and *Citrus sinensis*.

### 2.2. B. cinerea-Induced MnASIs Expression

**Figure 3:**
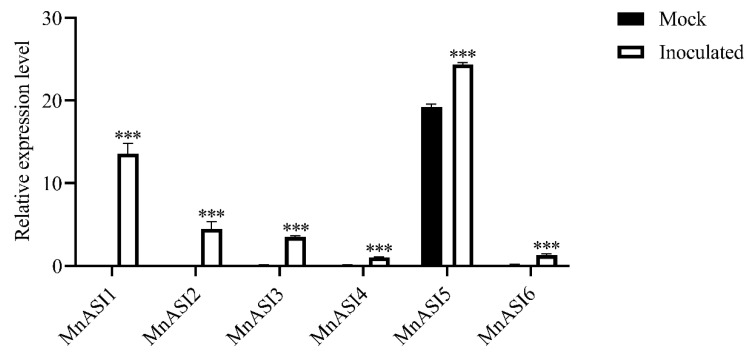
Relative expression levels of *MnASIs* in mock-treated (Mock) and *B. cinerea* inoculated (Inoculated) mulberry leaves. Error bars represent standard deviation, *n* = 3 (*** *p*-value < 0.001; two-tailed *t*-test).

The expression levels of six *MnASIs* in mulberry seedlings infected with *B. cinerea* were determined by qRT-PCR (Figure 3). The expression levels of all six *MnASIs* were significantly increased 3 days after inoculation, which was consistent with our previous transcriptome data (Table S1) [22]. These highly expressed α-amylase/subtilisin inhibitor genes may be involved in the resistance of mulberry to *B. cinerea*. *MnASI1* is the gene with the most increased expression after *B. cinerea* infection, which may play an important role in resistance to *B. cinerea* infection. In order to further verify the disease resistance function of *MnASIs*, *MnASI1* was selected for follow-up research.

### 2.3. Subcellular Localization of MnASI1

**Figure 4:**
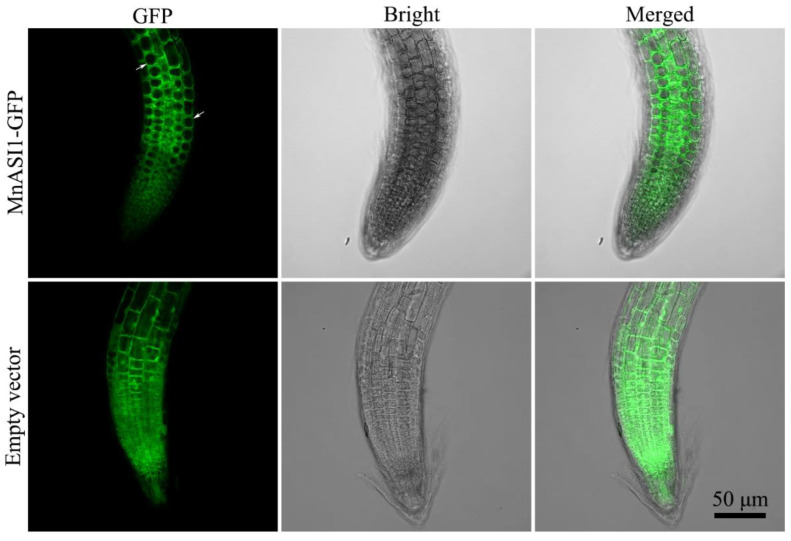
Image of Arabidopsis root tips producing *MnASI1*-GFP fusion protein. GFP fluorescence was detected by confocal laser scanning microscopy. The arrow shows the cell membrane.

Subcellular localization of transgenic *Arabidopsis* root tips was used in confocal microscopy (Figure 4). The results showed that the *MnASI1* protein was localized on the cell membrane, which indicated that *MnASI1* might play a disease-resistant role on the cell membrane.

### 2.4. Positive Regulation of MnASI1 for Resistance to B. cinerea

**Figure 5:**
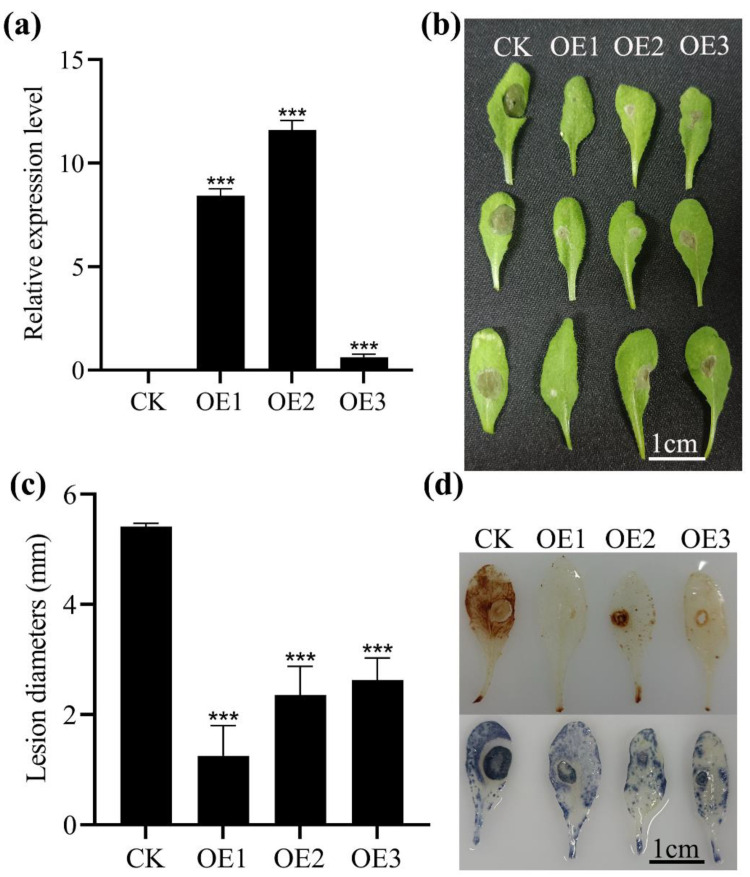
Resistance analysis of transgenic *Arabidopsis* to *B. cinerea*. (**a**) Relative expression levels of *MnASI1* in transgenic *Arabidopsis*. CK, empty vector transgenic; OE, *MnASI1* transgenic. (**b**) *Arabidopsis* leaves were photographed 36 h after infection with *B. cinerea*. (**c**) Quantitative analysis of resistance to *B. cinerea* in transgenic *Arabidopsis*. (**d**) DAB and NBT staining showed H_2_O_2_ and O_2_^−^ levels, respectively. Error bars represent standard deviation, *n* = 3 (*** *p*-value < 0.001; two-tailed *t*-test).

*Arabidopsis* was transformed with *MnASI1* cDNA to obtain three T_3_ transgenic lines (Figure 5). Afterward, the expression of *MnASI1* was confirmed by qRT-PCR in transgenic *Arabidopsis* (Figure 5a). To study the resistance of *Arabidopsis* transgenic with *MnASI1* to *B. cinerea*, the leaves of transgenic *Arabidopsis* were inoculated with an agar block containing *B. cinerea* hyphae (Figure 5b). Compared with severe lesions on control leaves 36 h after inoculation, only mild lesions appeared on leaves of *MnASI1* overexpression lines. Quantitative analysis revealed that *Arabidopsis* transformed with *MnASI1* inhibited the infection of *B. cinerea* (Figure 5c). Furthermore, the production of reactive oxygen species is a response to stress in plants. The contents of hydrogen peroxide (H_2_O_2_) and superoxide (O_2_^−^) in leaves were detected by DAB staining and NBT staining, respectively (Figure 5d). Compared with *Arabidopsis* transfected with *MnASI1*, large dark brown patches appeared after DAB staining in *Arabidopsis* transfected with empty vector, indicating the accumulation of H_2_O_2_, and large dark blue patches appeared after NBT staining, which was the accumulation of O_2_^−^.

### 2.5. Detection of Biochemical Indices

**Figure 6:**
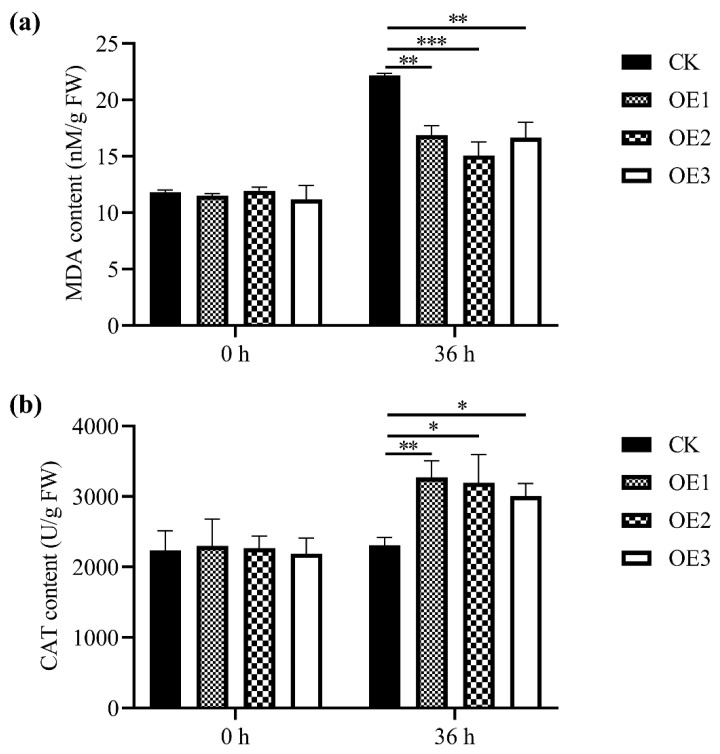
Detection of physicochemical indexes before and after inoculation of *B. cinerea*. (**a**) Malondialdehyde (MDA) content. (**b**) Catalase (CAT) activity. CK, empty vector transgenic; OE, *MnASI1* transgenic. Error bars represent standard deviation, *n* = 3 (* *p*-value < 0.05, ** *p*-value < 0.01, and *** *p*-value < 0.001; two-tailed *t*-test).

To determine the physiological changes of transgenic *Arabidopsis*, MDA content and CAT activity were measured (Figure 6). There was no significant difference in MDA content between *MnASI1* transgenic *Arabidopsis* and empty vector transgenic *Arabidopsis* before *B. cinerea* infection (Figure 6a). After 36 h infection with *B. cinerea*, the MDA content of transgenic *Arabidopsis* increased, while the MDA content of *MnASI1* transgenic *Arabidopsis* was significantly lower than that of empty vector transgenic *Arabidopsis*. These results indicated that the plasma membrane damage of empty vector transgenic *Arabidopsis* was more severe than that of *MnASI1* transgenic *Arabidopsis*. Similarly, there was no significant difference in CAT activity between *MnASI1* and empty vector transgenic *Arabidopsis* before infection with *B. cinerea* (Figure 6b). After 36 h infection with *B. cinerea*, the CAT activity of transgenic *Arabidopsis* increased, while the CAT content of *MnASI1* transgenic *Arabidopsis* was significantly higher than that of empty vector transgenic *Arabidopsis*. These results suggest that overexpression of *MnASI1* enhanced plant resistance to oxidative damage.

**Figure 7:**
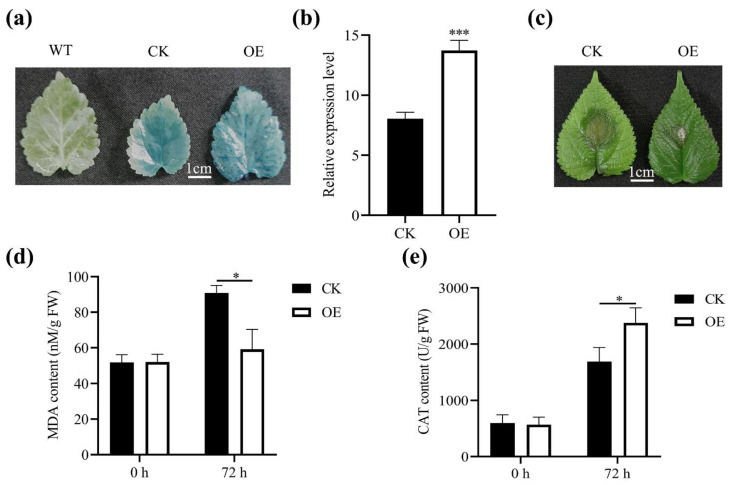
Analysis of *MnASI1* resistance to *B. cinerea* using mulberry transient expression. (**a**) GUS staining of untreated and transiently expressed mulberry leaves. (**b**) Expression analysis of *MnASI1* in transiently expressed mulberry leaves. (**c**) Mulberry leaves were photographed 72 h after infection with *B. cinerea*. (**d**) Malondialdehyde (MDA) content. (**e**) Catalase (CAT) activity. CK, empty vector transgenic; OE, *MnASI1* transgenic. Error bars represent standard deviation, *n* = 3 (* *p*-value < 0.05 and *** *p*-value < 0.001; two-tailed *t*-test).

To investigate the role of *MnASI1* in mulberry, transient overexpression was performed in mulberry (Figure 7). The beta-glucuronidase (GUS) histochemical analysis showed strong GUS staining in the leaves of mulberry seedlings, indicating that this transient expression system is effective in mulberry (Figure 7a). The expression levels of *MnASI1* were up-regulated in mulberry leaves overexpressing *MnASI1* compared with those overexpressing empty vectors (Figure 7b). The resistance of mulberry overexpressing *MnASI1* to *B. cinerea* was enhanced compared with the overexpressing empty vector (Figure 7c). Under the condition of *B. cinerea* infection, the transient expression of *MnASI1* significantly reduced the content of MDA in mulberry seedlings (Figure 7d) and increased the content of CAT (Figure 7e), which was consistent with the previous results of *MnASI1* transgenic *Arabidopsis*.

### 2.6. MnASI1 Transgenic Plants Enhance PR1 Expression

**Figure 8:**
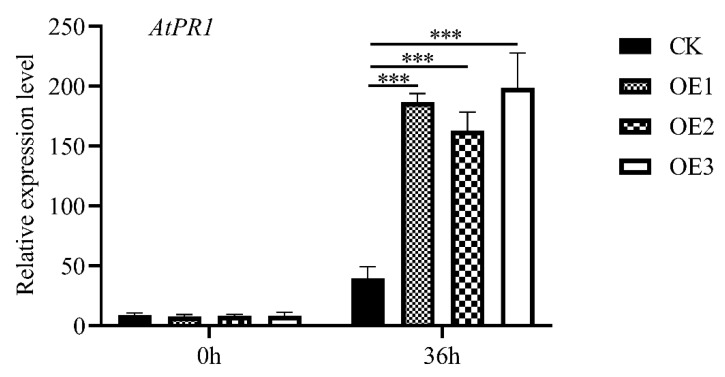
Relative expression of *AtPR1* gene in transgenic *Arabidopsis* before and after inoculation with *B. cinerea*. CK, empty vector transgenic; OE, *MnASI1* transgenic. Error bars represent standard deviation, *n* = 3 (*** *p*-value < 0.001; two-tailed *t*-test).

*PR1* is a plant defense-related marker gene. The results showed that the expression of *AtPR1* in empty vector and *MnASI1* transgenic *Arabidopsis* was up-regulated 36 h after *B. cinerea* infection, and empty vector transgenic *Arabidopsis* was significantly lower than *MnASI1* transgenic *Arabidopsis* (Figure 8). This indicated that *MnASI1* overexpression in *Arabidopsis* could enhance the resistance to *B. cinerea* by inducing the expression of the resistance-related gene.

## 3. Discussions

Many resistant plants have evolved specific PIs that both regulate plant protease activity and promote plant defense against pests and pathogens [23]. As a class of PIs, there are few studies on plant α-amylase/subtilin inhibitors genes, and most reports have not performed functional analysis. Previous studies related to ASIs disease resistance mainly focused on the study of recombinant ASIs protein. For example, after infection with *P. palmivora* spores pretreated with recombinant *HbASI*, the growth, lesion number, and scopolamine content of the spores were low, and the effect was better with the increase of recombinant *HbASI* content [2]. Similarly, Kunitz inhibitory fusion protein had a certain inhibitory effect on the growth of *F. moniliforme* mycelium [24]. The study of *MnASIs* gene in mulberry will help to further understand the characteristics and function of this gene. Based on amino acid sequence features (Figure 1), *MnASIs* can be classified as α-amylase/subtilisin inhibitors, belonging to group C of the kunitz protease inhibitor family [25]. Furthermore, phylogenetic tree results indicated that *MnASIs* clustered with other plant α-amylase/subtilin inhibitors (Figure 2). High homology between *MnASIs* and *V. vinifera* a-amylase/subtilisin inhibitors. Analysis showed that the mulberry *MnASIs* were intronless (Table 1). These results are consistent with previous reports on intronless PI genes [26]. Intronless genes may be a structural feature that provides a selective advantage to rapidly encode and flip transcripts in response to a variety of exogenous signals without significant delays [27].

In plants, the expression level of pathogenicity-related (PR) genes is generally low, and only high induced expression during pathogen infection [28,29,30]. Similar to PR genes, except for *MnASI5*, the other five *MnASIs* were only induced to express during *B. cinerea* infection (Figure 3). Inoculation of mulberry leaves with *B. cinerea* increased the expression of *MnASIs*, indicating that *MnASIs* are induced by biological elicitors and participate in plant defense responses. These results are consistent with previous reports [2,9]. The *MnASI5* gene was highly expressed in mulberry leaves mock-treated and *B. cinerea* inoculated. This suggests that the *MnASI5* gene may have dual functions of development and self-protection. The transcriptome data were consistent with the qRT-PCR results as a whole (Figure 3 and Table S1), and the expression of *MnASIs* was significantly enhanced, but the multiple of *MnASIs* expression enhancement was different, which may be caused by mulberry individual differences.

To further study the role of *MnASIs* in disease resistance, the empty vector and *MnASI1* gene transgenic *Arabidopsis* and mulberry transiently overexpressed were constructed (Figure 5 and Figure 7). Compared with plants transformed with empty vector, after inoculation with *B. cinerea*, plants transformed with *MnASI1* gene had smaller necrotic spots. The results showed that molecular and physiological responses to *B. cinerea* included ROS production and transcriptional responses. A more significant response of the SA-mediated defense gene was detected in *B. cinerea*-infected transgenic *MnASI1* plants (Figure 8), suggesting that overexpression of *MnASI1* enhances the defense capacity of transgenic plants by activating hypersensitive responses. The SA-dependent signaling pathway leads to the expression of the PR protein *AtPR1*, which promotes resistance. Consistent with previous reports, the SA signaling pathway plays an important role in response to *B. cinerea* infection [31]. *Arabidopsis* had obvious disease at 36 h of *B. cinerea* infection, while mulberry had obvious disease at 72 h of *B. cinerea* infection, indicating that mulberry was more resistant to *B. cinerea*.

MDA is a key lipid peroxidation product in plant defense [32]. The results showed that transgenic *MnASI1* plants resulted in a decrease in MDA content (Figure 6a and Figure 7d). MDA content in plants is often associated with oxidative stress. Transfection of *MnASI1* can reduce cell membrane damage. When plants are infected with pathogens, the activity of plant defense-related enzymes is induced, which helps scavenge peroxides [33]. The CAT activity of *MnASI1* overexpressing plants was significantly enhanced (Figure 6b and Figure 7e), thus resisting *B. cinerea* infection.

Previous studies have shown that ASI recombinant protein has a disease-resistance effect, and this study is the first to report the disease-resistance mechanism of the ASI gene in mulberry, indicating that ASI has a disease-resistance effect both in vitro and in vivo. However, the upstream regulatory genes and downstream interacting genes of ASI are still unclear and need to be further studied. In addition, alginate encapsulation of plant biocontrol bacteria has been studied to some extent [34], and ASI protein may also be coated with alginate for plant disease control in the future.

## 4. Materials and Methods

### 4.1. Phylogenetic Tree of ASIs

To investigate evolutionary relationships, the full-length amino acid sequences of ASIs proteins were aligned using ClustalW under default settings, and a neighborhood-joining phylogenetic tree of ASIs was subsequently constructed using MEGA 6 [35]. Bootstrap analysis of 1000 replicates was performed.

### 4.2. Quantitative Real-Time PCR

Total RNA was extracted using RNAiso Plus Kit (Takara Bio., Kusatsu, Shiga, Japan). cDNA synthesis using the PrimeScript™ RT Reagent Kit (Takara Bio., Kusatsu, Shiga, Japan). qRT-PCR detection was performed using SYBR^®^ Premix Ex Taq™ II (Takara Bio., Kusatsu, Shiga, Japan) and StepOnePlus™ Real-time PCR system (Applied Biosystems, Waltham, MA, USA). The actin gene was used as an internal reference gene. qRT-PCR was performed in three technical replicates. qRT-PCR primers were shown in Table S2.

### 4.3. Transformation of Arabidopsis

To generate overexpression plasmid, *MnASI1* was cloned into the *Kpn*I (5′-GGGTACCATGGCTTCTCGTGGCATGGCAG-3′) and *Sal*I (5′-GCGTCGACTTATATTGTAGCTCGCTCAAACA-3′) restriction sites of the pLGNL vector and transformed into the *A. tumefaciens* GV3101. In subcellular localization, insert the *MnASI1* gene into the *Kpn*I (5′-GGGTACCATGGCTTCTCGTGGCATGGCAG-3′) and *BamH*I (5′-CGGGATCCTATTGTAGCTCGCTCAAACA-3′) sites of the pZYGC expression vector, which includes a green fluorescent protein (GFP). Then pLGNL-*MnASI1* and pZYGC-*MnASI1* were transferred into *Arabidopsis* (Col-0) by flower dip method [36].

### 4.4. Transient Expression Analysis of Mulberry Gene Function

The *A. tumefaciens* GV3101 containing pLGNL-*MnASI1* or pLGNL vectors prepared with transformation solution (1/2 MS, 5% sucrose, 200 µM acetosyringone and 0.05% Tween-20, pH 5.6) to a final OD_600_ to 0.5. The 15-day-old mulberry seedlings were placed in the transformation solution of the GV3101 containing pLGNL-*MnASI1* or pLGNL, and vacuumed at room temperature for 20 min.

### 4.5. Resistance Analysis of Transgenic Arabidopsis and Mulberry to B. cinerea

The hyphal fragments were placed on plant leaves. Photographs were taken 36 h after inoculation. The plants transformed into pLGNL were used as controls. Malondialdehyde (MDA) content and catalase (CAT) activity were determined using a Malondialdehyde Assay Kit (Solarbio, Beijing, China) and a Catalase Assay Kit (Solarbio, Beijing, China) according to the manufacturer’s instructions. All treatments were repeated three times. The content of superoxide radical (O_2_^−^) and hydrogen peroxide (H_2_O_2_) in leaves was determined by nitroblue tetrazole (NBT) and 3,3′-diaminobenzidine (DAB) staining as described previously [37,38].

### 4.6. Statistical Analysis 

All data were subjected to Student’s *t*-test or one-way ANOVA with SPSS 26.0 software. These values are expressed as mean ± standard deviation (SD). *p* < 0.05 was considered statistically significant.

## Figures and Tables

**Table 1:** Characterization of α-amylase/subtilisin inhibitors in *M. notabilis*.

Gene Name	Gene Name	GenBank Acc.	CDS (bp)	Size (aa)	MW (kDa)	Predicted pI
*MnASI1*	*L484_010983*	EXB74706.1	576	191	20.82	4.46
*MnASI2*	*L484_010984*	EXB74707.1	603	200	21.7	5.41
*MnASI3*	*L484_010986*	EXB74709.1	624	207	22.65	8.35
*MnASI4*	*L484_010988*	EXB74711.1	579	192	21.19	7.52
*MnASI5*	*L484_010987*	EXB74710.1	576	191	21.12	4.93
*MnASI6*	*L484_010989*	EXB74712.1	579	192	21.24	8.53

## Data Availability

All data supporting the findings of this study are available within the paper and its supplementary data are published online.

## Supplementary Materials

The following supporting information can be downloaded at https://www.mdpi.com/article/10.3390/ijms232113372/s1.
