# Supplementary material for: MnASI1 Mediates Resistance to Botrytis cinerea in Mulberry (Morus notabilis)
Source: Int J Mol Sci. 2022 Nov 2;23(21):13372. doi: 10.3390/ijms232113372 (PMC9656013; doi:10.3390/ijms232113372)
Supplement: Supplementary file 1 [file ijms-23-13372-s001.zip › ijms-1994370 -supplyment Table S1.pdf]

Table S1. Differential expression analysis of *MnASI* gene in Mock and Inoculated.

Gene ID	Inoculated FPKM	Mock FPKM	log2(Inoculated/Mock)
MnASI1	6310.57	66.92	6.556122
MnASI2	2341.096	3.676	9.299648
MnASI3	1939.94	487.78	1.988951
MnASI4	601.136	79.293	2.909071
MnASI5	7591.113	4709.027	0.688883
MnASI6	2.46	0	6.714959
