# Supplementary material for: MnASI1 Mediates Resistance to Botrytis cinerea in Mulberry (Morus notabilis)
Source: Int J Mol Sci. 2022 Nov 2;23(21):13372. doi: 10.3390/ijms232113372 (PMC9656013; doi:10.3390/ijms232113372)
Supplement: Supplementary file 1 [file ijms-23-13372-s001.zip › Table S2.pdf]

Table S2. Primers for real-time PCR.

Gene symbol	Forward primer	Reverse primer
MnASI1	GCTCGTGCCCGTTCTATGT	TCCCGTCCCAATCAATCTG
MnASI2	ACGGCTCGTGTCCTTTCTA	GTATCTCAGGTCGTCTCCC
MnASI3	ACGACCCGTGCCCACTCTAC	CCGCCAACCTTTGTCCATTT
MnASI4	TGAGGGAGACGAGGGACT	CTACAACCAGGGCAAGCA
MnASI5	GATGGCGAACTTATCAGCACC	AACTTGCCCACATCTGAACCA
MnASI6	GGGAGACGAGGGACTTCAA	GAGCAGCCCAGACCAACAA
MnActin	GCATGAAGATCAAGGTGGTG	CATCTGCTGGAAGGTGCTAA
AtPR1	GCAGAACAACTAAGAGGCAA	CAGCGTAGTTGTAGTTAGCC
AtActin	TGCTGAGCTTATCGATTCCG	TTCGGTGATGGGAATACAG
